# Supplementary material for: Identification of photoperiod-induced specific miRNAs in the adrenal glands of Sunite sheep (Ovis aries)
Source: Front Vet Sci. 2022 Jul 22;9:888207. doi: 10.3389/fvets.2022.888207 (PMC9354845; doi:10.3389/fvets.2022.888207)
Supplement: Supplementary file 1 [file Data_Sheet_1.ZIP › Supplementary file/miRDeep2 file.zip!/miRDeep2 file/Novel_Structure/LP42A2/Novel_445.pdf]

| | | |
|---|---|---|
| Provisional ID | : | NC_019458.2_2482 |
| Score total | : | 2.3 |
| Score for star read(s) | : | -1.3 |
| Score for read counts | : | 0 |
| Score for mfe | : | 2 |
| Score for randfold | : | 1.6 |
| Score for cons. seed | : | |
| Total read count | : | 12 |
| Mature read count | : | 12 |
| Loop read count | : | 0 |
| Star read count | : | 0 |

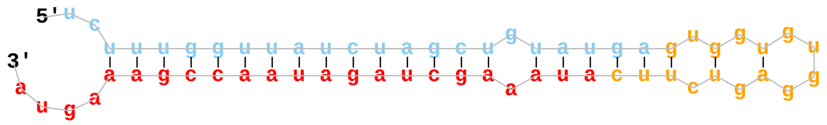


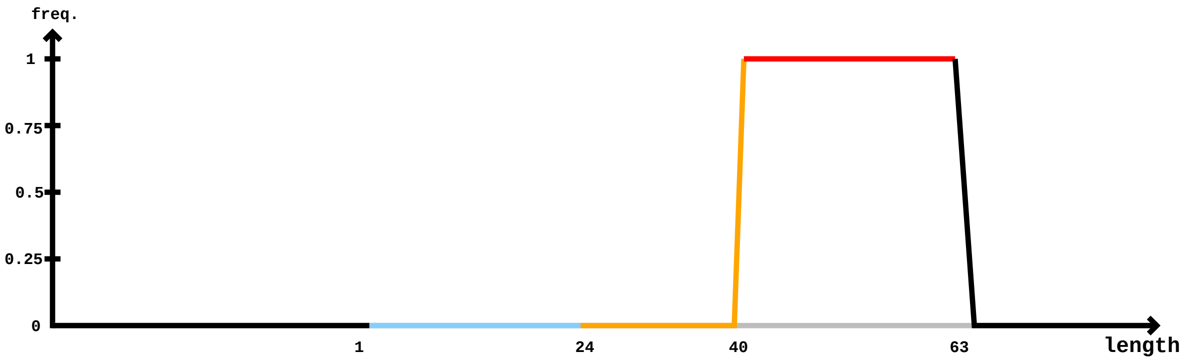


Star Mature

```
5'- gacugcagccaggaggcggguugguuguuaucuuugguuaucuagcuguaugaguggugugguggagucuucauaaagcuagauaaccgaaaguaaaaauaaccccauacacugc   -3'    exp
    .......(((....)))(((((((....((((.(((((((((((((((.((((((.(.(....).).)))))).))))))))))))))).))))...))))))).........    reads      mm          sample
    .....................................................................auaaagcuagauaaccgaaagua....................    12         0           seq
```
